# Antibody responses to the BNT162b2 mRNA vaccine in individuals previously infected with SARS-CoV-2

**DOI:** 10.1038/s41591-021-01325-6

**Published:** 2021-04-01

**Authors:** Joseph E. Ebinger, Justyna Fert-Bober, Ignat Printsev, Min Wu, Nancy Sun, John C. Prostko, Edwin C. Frias, James L. Stewart, Jennifer E. Van Eyk, Jonathan G. Braun, Susan Cheng, Kimia Sobhani

**Affiliations:** 1 Department of Cardiology, Smidt Heart Institute, Cedars-Sinai Medical Center, Los Angeles, CA USA; 2 Advanced Clinical Biosystems Institute, Department of Biomedical Sciences, Cedars-Sinai Medical Center, Los Angeles, CA USA; 3 F. Widjaja Foundation Inflammatory Bowel and Immunobiology Research Institute, Cedars-Sinai Medical Center, Los Angeles, CA USA; 4 Applied Research and Technology, Abbott Diagnostics, Abbott Park, IL USA; 5 Department of Pathology and Laboratory Medicine, Cedars-Sinai Medical Center, Los Angeles, CA USA

**Keywords:** Viral infection, RNA vaccines, SARS-CoV-2, Antibodies

## Abstract

In a cohort of BNT162b2 (Pfizer–BioNTech) mRNA vaccine recipients (*n* = 1,090), we observed that spike-specific IgG antibody levels and ACE2 antibody binding inhibition responses elicited by a single vaccine dose in individuals with prior SARS-CoV-2 infection (*n* = 35) were similar to those seen after two doses of vaccine in individuals without prior infection (*n* = 228). Post-vaccine symptoms were more prominent for those with prior infection after the first dose, but symptomology was similar between groups after the second dose.

## Abstract (web summary)

Virus-specific antibody levels after a single dose of the BNT162b2 vaccine in individuals previously infected with SARS-CoV-2 are similar to levels after two doses of the vaccine in infection-naive individuals.

## Main

Messenger RNA (mRNA) vaccines against severe acute respiratory syndrome coronavirus 2 (SARS-CoV-2), the causative agent of Coronavirus Disease 2019 (COVID-19), offer great promise for curbing the spread of infection^1–3^. Challenges to the supply chain have prompted queries around whether single-dose administration rather than double-dose administration might suffice for certain individuals, including those recovered from prior infection. Emerging immune data, including detectable presence of anti-SARS-CoV-2 antibodies and virus-specific T cells, have suggested possible alternate vaccination strategies for previously infected individuals^4–6^. Recent small studies have indicated that individuals with prior infection might have naturally acquired immunity that could be sufficiently enhanced by a single dose rather than a double dose of administered vaccine^7,8^. To this end, we evaluated SARS-CoV-2-specific antibody responses after the first and second doses of the BNT162b2 (Pfizer–BioNTech) mRNA vaccine in a large and diverse cohort of healthcare workers. We compared the responses of individuals with confirmed prior infection to those of individuals without prior evidence of infection.

We enrolled healthcare workers from across a large academic medical center in Southern California. Vaccine recipients (*n* = 1,090) who provided at least one blood sample for antibody testing were aged 41.9 ± 12.2 years and were 60.7% female and 53.3% non-White (Table 1): 981 vaccine recipients, including 78 with prior SARS-CoV-2 infection, provided baseline (pre-vaccine) samples; 525 (35 with prior infection) provided samples after dose 1; and 239 (11 with prior infection) provided samples after dose 2. A total of 217 individuals (ten with prior infection) provided blood samples at all three time points. Antibody levels were measured at three time points: before or up to 3 d after dose 1; within 7–21 d after dose 1; and within 7–21 d after dose 2. Because the timing of the first blood draw for antibody testing could confound the association of spike glycoprotein-specific IgG (IgG(S-receptor-binding domain (RBD))) with prior infection versus early vaccine response^9^, we used nucleoprotein-specific IgG (IgG(N)) to denote prior SARS-CoV-2 exposure while recognizing a small potential for representing cross-reactivity with other coronaviruses. Given that the BNT162b2 vaccine delivers mRNA encoding only for spike protein, the expected elicited response is production of IgG(S-RBD) antibodies and not IgG(N) antibodies;^10^ furthermore, IgG(N) antibodies are also known to represent a durable marker and indicator of post-infectious status^11^. Accordingly, we determined prior SARS-CoV-2 infection status and timing in relation to the first vaccine date, based on concordance of data documented in health records, presence of any IgG(N) antibodies at baseline pre-vaccination testing and the self-reported survey information collected. All cases of data discrepancy regarding prior SARS-CoV-2 infection status underwent manual physician adjudication, including medical chart review for evidence of positive SARS-CoV-2 polymerase chain reaction (PCR) or antibody testing that could have been performed by outside institutions or otherwise documented in the medical record.

**Table 1:** Characteristics of the study cohort

	Total	Pre-vaccine	Post-vaccine dose 1	Post-vaccine dose 2
*n*	1,090	981	525	239
Age in years, mean (s.d.)	41.89 (12.18)	41.60 (12.05)	43.66 (12.79)	44.12 (12.65)
Race, *n* (%)				
White	509 (46.7)	453 (46.2)	263 (50.1)	130 (54.4)
Black or African American	36 (3.3)	33 (3.4)	22 (4.2)	9 (3.8)
Asian	300 (27.5)	265 (27.0)	154 (29.3)	67 (28.0)
Native Hawaiian/Pacific Islander	29 (2.7)	27 (2.8)	14 (2.7)	3 (1.3)
American Indian/Alaska Native	2 (0.2)	2 (0.2)	0 (0.0)	0 (0.0)
Multiple/other	139 (12.8)	130 (13.2)	58 (11.1)	27 (11.4)
Prefer not to answer	75 (6.9)	71 (7.2)	14 (2.6)	3 (1.3)
Ethnicity, *n* (%)				
Hispanic/Latinx	139 (12.8)	126 (12.8)	55 (10.5)	20 (8.4)
Non-Hispanic/Latinx	881 (80.8)	788 (80.3)	460 (87.6)	216 (90.4)
Prefer not to answer	70 (6.4)	67 (6.8)	10 (1.9)	3 (1.3)
Sex, *n* (%)				
Male	362 (33.2)	331 (33.7)	159 (30.3)	65 (27.2)
Female	662 (60.7)	587 (59.8)	353 (67.2)	168 (70.3)
Other	1 (0.1)	1 (0.1)	1 (0.2)	1 (0.4)
Prefer not to answer	65 (6.0)	62 (6.3)	12 (2.3)	5 (2.1)
Prior SARS-CoV-2 infection, *n* (%)	86 (7.9)	78 (8.0)	35 (6.7)	11 (4.6)
Antibody levels, mean (%)				
Architect IgG index (S/C) (IgG(N))	0.30 (0.86)	0.25 (0.84)	0.36 (0.90)	0.34 (0.82)
Architect IgM index (S/C)	0.99 (2.41)	0.26 (1.24)	2.11 (4.11)	3.38 (5.96)
Architect Quant IgG II (AU ml^−1^) (IgG(S-RBD))	2,801.04 (6,159.27)	103.90 (693.89)	3,183.38 (7,299.73)	24,084.06 (16,367.63)

**Fig. 1:**
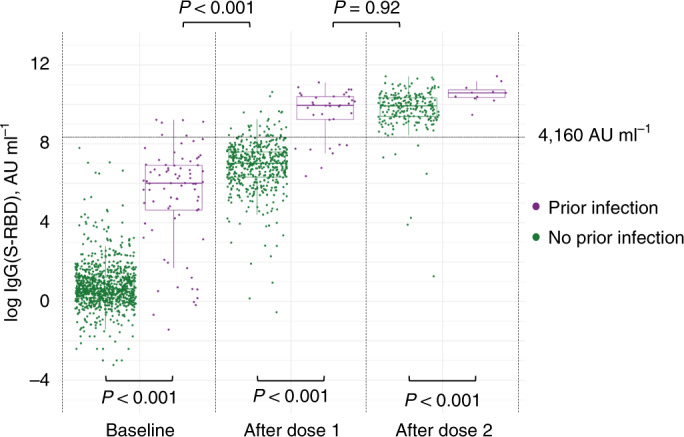
IgG(S-RBD) antibody response to mRNA SARS-CoV-2 vaccination in individuals with and without prior SARS-CoV-2 infection. Box plots display the median values with the interquartile range (lower and upper hinge) and ±1.5-fold the interquartile range from the first and third quartile (lower and upper whiskers). We used two-sided Wilcoxon tests, without adjustment for multiple testing, to perform the following between-group comparisons: (1) infection-naive individuals (*n* = 903) and those with prior infection (*n* = 78), both at baseline (*P* < 0.001); (2) infection-naive individuals (n = 490) and those with prior infection (*n* = 35), both after dose 1 (*P* < 0.001); (3) infection-naive individuals (*n* = 228) and those with prior infection (*n* = 11), both after dose 2 (*P* < 0.001); (4) infection-naive individuals (*n* = 490) after dose 1 and those with prior infection (*n* = 78), both at baseline (*P* < 0.001); and (5) infection-naive individuals (*n* = 228) after dose 2 and those with prior infection (*n* = 35) after dose 1 (*P* = 0.92).

**Extended Data Fig. 1:**
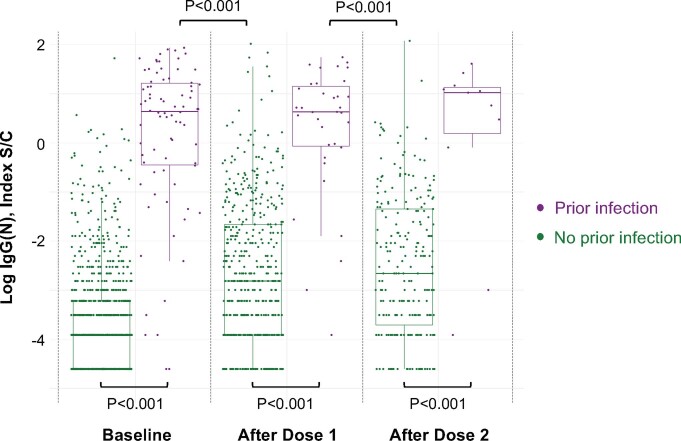
Anti-Nucleocapsid Protein IgG Antibody Response to mRNA SARS-CoV-2 Vaccination in Persons With and Without Prior SARS-CoV-2 infection. Box plots display the median values with the interquartile range (lower and upper hinge) and ±1.5-fold the interquartile range from the first and third quartile (lower and upper whiskers). We used two-sided Wilcoxon tests, without adjustment for multiple testing, to conduct the following between-group comparisons: (1) infection naïve (n = 903) and those with prior infection (n = 78), both at baseline (P < 0.001); (2) infection naïve (n = 490) and those with prior infection (n = 35), both following dose 1 (P < 0.001); (3) infection naïve (n = 228) and those with prior infection (n = 11), both following dose 2 (P < 0.001); (4) infection naïve (n = 490) following dose 1 and those with prior infection (n = 78) at baseline (P < 0.001); and, (5) infection naïve (n = 228) following dose 2 and those with prior infection (n = 35) following dose 1 (P < 0.001).

For both IgG(N) (representing response to prior infection) and IgG(S-RBD) (representing response to either prior infection or vaccine), as expected, individuals with prior SARS-CoV-2 infection had higher antibody levels at all time points (*P* ≤ 0.001) (Supplementary Tables 1 and 2 and Extended Data Fig. 1). Notably, IgG(S-RBD) levels were only slightly lower in previously infected individuals at baseline when compared to infection-naive individuals who had received a single vaccine dose (log-median AU ml^−1^ (interquartile range, 6.0 (4.6, 6.9) versus 7.0 (6.3, 7.6)), *P* < 0.001). Moreover, IgG(S-RBD) levels were not significantly different among previously infected individuals after a single dose and infection-naive individuals who had received two doses (10.0 (9.2, 10.4) versus 9.9 (9.4, 10.3), *P* = 0.92) (Fig. 1). Similar results were found in a sensitivity analysis including only individuals who had antibody immunoassays performed at all three time points (Supplementary Tables 3 and 4). Specifically, those with prior infection had higher IgG(S-RBD) than those without prior infection at all time points. No difference in IgG(S-RBD) levels was detected between those with prior infection after one dose of vaccine and those without prior infection after two doses (10.2 (8.4, 10.5) versus 9.9 (9.4, 10.3), *P* = 0.58).

**Extended Data Fig. 2:**
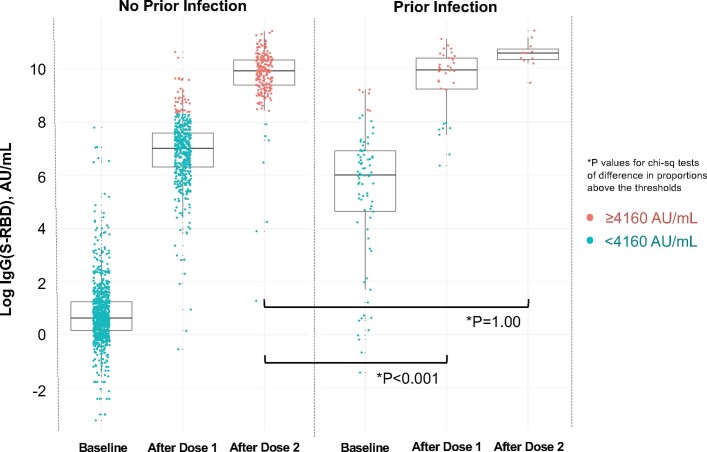
Anti-Spike Receptor Binding Domain IgG Antibody Response to mRNA SARS-CoV-2 Vaccination in Persons With and Without Prior SARS-CoV-2 infection: Values Above and Below 4160 AU/mL. Box plots display the median values with the interquartile range (lower and upper hinge) and ±1.5-fold the interquartile range from the first and third quartile (lower and upper whiskers). We used two-sided Chi-Square tests with the Yate’s correction, without adjustment for multiple testing, to conduct the following between-group comparisons: (1) infection naïve (n = 221) following dose 2 and those with prior infection (n = 27) following dose 1 (P < 0.001); and, (2) infection naïve (n = 221) and those with prior infection (n = 11), both following dose 2 (P = 1.00).

**Extended Data Fig. 3:**
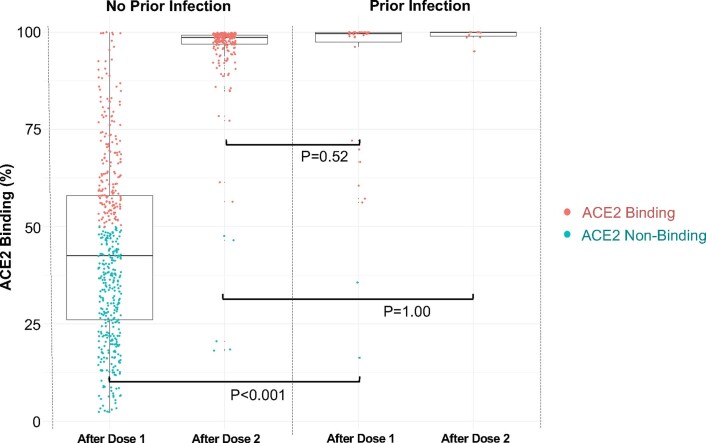
ACE2 Antibody Binding Capacity in Persons With and Without Prior SARS-CoV-2 infection: Values Above and Below 50%. Box plots display the median values with the interquartile range (lower and upper hinge) and ±1.5-fold the interquartile range from the first and third quartile (lower and upper whiskers). Colors denote values above and below the 50% neutralizing threshold. We used two-sided Wilcoxon tests, without adjustment for multiple testing, to conduct the following between-group comparisons: (1) infection naïve (n = 490) and those with prior infection (n = 35), both following dose 1 (P < 0.001); (2) infection naïve (n = 228) and those with prior infection (n = 11), both following dose 2 (P = 1.00); and, (3) infection naïve (n = 228) following dose 2 and those with prior infection (n = 35) following dose 1 (P = 0.52).

For surrogate measures of antibody neutralization, we examined IgG(S-RBD) levels at or above 4,160 AU ml^−1^ given that this threshold corresponds to a 0.95 probability of obtaining a plaque reduction neutralization test (PRNT) ID_50_ at a 1:250 dilution. Proportions of IgG(S-RBD) ≥ 4,160 AU ml^−1^ were similar between previously infected individuals at baseline compared to infection-naive individuals after a single dose (*P* = 1.00). Notably, these proportions were lower in previously infected individuals after a single dose than in infection-naive individuals after two doses (*P* < 0.001); there were no between-group differences after two doses (Supplementary Table 5 and Extended Data Fig. 2). We also used an angiotensin-converting enzyme 2 (ACE2) binding inhibition assay that correlates well with the SARS-CoV-2 PRNT methodology and exhibits a high correlation with the IgG(S-RBD) assay threshold (*r*^2^ = 0.95). We found that ACE2 binding inhibition was significantly higher among previously infected individuals than infection-naive individuals after a single vaccine dose (94.3% versus 37.3%, *P* < 0.001), with no between-group difference seen after the second dose (100.0% versus 97.8%, *P* = 1.00). In time-shifted analyses, there was no difference in ACE2 binding between individuals with prior SARS-CoV-2 infection after a single dose and infection-naive individuals after two doses (94.3% versus 97.8%, *P* = 0.52) (Supplementary Table 6 and Extended Data Fig. 3).

**Extended Data Fig. 4:**
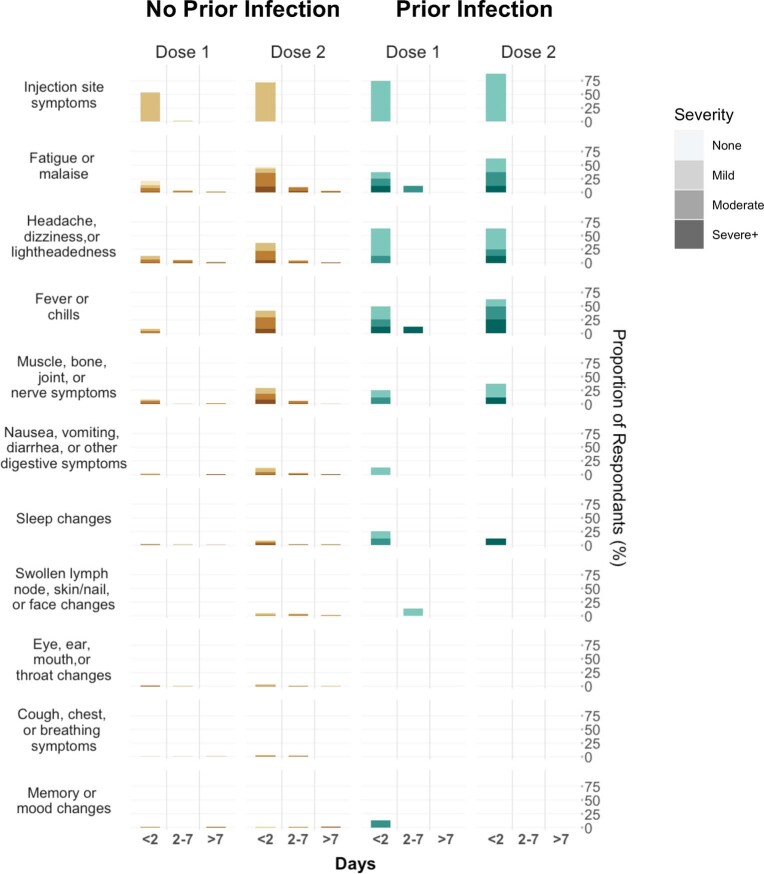
Post-Vaccination Symptoms in Persons With and Without Prior SARS-CoV-2 infection. Bar graphs display the frequency and severity of symptoms reported following each dose of vaccine administered, by prior infection status.

In parallel with antibody response analyses, we also examined post-vaccine symptomology (Supplementary Tables 7–11 and Extended Data Fig. 4). We observed that previously infected individuals experienced significant post-vaccine symptoms (that is, reactogenicity) more frequently than infection-naive individuals after dose 1 (36.8% versus 25.0%, *P* = 0.03). However, there was no between-group difference in significant symptoms after dose 2 (51.3% versus 58.7%, *P* = 0.26). In time-shifted analyses, infection-naive individuals had higher reactogenicity after dose 2 than previously infected individuals after their first dose (58.7% versus 36.8%, *P* < 0.001). Fever and chills were more common among previously infected vaccine recipients after the first dose, whereas infection-naive individuals were more likely to experience headache, dizziness or lightheadedness after the second dose. In analyses of changes from dose 1 to dose 2, reactogenicity increased in frequency for infection-naive individuals (25.0% versus 58.7%, *P* < 0.001) and less so in previously infected individuals (36.8% versus 51.3%, *P* = 0.08).

Overall, we found that individuals previously infected with SARS-CoV-2 developed vaccine-induced antibody responses after a single dose of the BNT162b2 (Pfizer–BioNTech) mRNA vaccine that were similar to antibody responses seen after a two-dose vaccination course administered to infection-naive individuals. Our findings in a large and diverse cohort of healthcare workers expand from the results of smaller studies that have indicated higher levels of anti-S antibodies at baseline, and after a single mRNA vaccine dose, in previously infected individuals compared to those without prior infection^7,8,12,13^. In a total cohort of over 1,000 vaccine recipients, including several hundred with blood sampling after administered vaccine doses, we found that the anti-S antibody response after a single vaccine dose in previously infected individuals was similar to the response seen after two doses in all vaccine recipients irrespective of prior infection status. We further assessed the neutralization potential of elicited antibodies using a high-throughput ACE2 inhibition neutralization surrogate assay. Similarly to findings from a smaller study that directly measured antibody neutralizing capacity in 59 volunteers^8^, we found, in our large cohort, that a second vaccine dose did not offer previously infected individuals a substantially greater benefit over a single dose in antibody neutralizing potential. Thus, our data suggest that a single dose of the Pfizer–BioNTech vaccine is sufficient for individuals with prior SARS-CoV-2 infection, not only when considering the response in anti-S antibody levels but also when examining results of an ACE2 inhibition assay indicating the potential neutralizing capability of elicited antibodies.

Limitations of our study include the 21-d timeframe within which antibodies were measured after each vaccine dose. Longer-term follow-up can provide additional information regarding the putative duration of immunity acquired from receiving a single dose versus a double dose of vaccine. Measures of T cell responses can shed further light on how a single dose versus a double dose of vaccine might be sufficient for augmenting T cell memory in previously infected individuals^12^. Further studies are needed to determine if a certain window for vaccination timing might be optimal to maximize efficacy as well as safety in previously infected individuals. Larger-sized cohorts are needed for sufficient statistical power to examine differences across demographic and clinical subgroups that are known to exhibit variation in antibody responses after vaccination^14–16^. When neutralizing capacity was estimated using a conservative IgG(S-RBD) threshold of >4,160 AU ml^−1^, the single-dose response in previously infected individuals was numerically similar, albeit statistically significantly lower, than the antibody response after two doses in infection-naive individuals. When applying this conservative threshold of >4,160 AU ml^−1^, which correlates with a 95% probability of high neutralizing antibody titer^17^, statistical comparisons are susceptible to the influence of extreme values in the context of smaller-sized subgroups. Notably, there was no significant difference in the surrogate measure of ACE2 binding inhibition between persons with and without prior infection in time-shifted analysis after vaccine dose 1 and dose 2, respectively. Notwithstanding methodological differences between examining IgG(S-RBD) levels and assays of ACE2 binding inhibition, these surrogate measures suggest materially similar levels of achieved neutralization capacity. Some variation in antibody responses might also be related to heterogeneity within previously infected individuals, including in timing and severity of prior illness. Although circulating antibody levels alone are not definitive measures of immune status, serial measures of the serological response to either natural or inoculated exposures are known to correlate well with effective protective immunity^18^, and our results indicate their potential utility in guiding vaccine deployment strategies for both previously infected and infection-naive individuals.

Our results offer preliminary evidence in support of a middle ground between public health-motivated and immunologically supported vaccine strategies. If validated, an approach that involves providing a single dose of vaccine to individuals with a confirmed history of SARS-CoV-2 infection along with an on-time complete vaccine schedule for infection-naive individuals could maximize the benefit of a limited vaccine supply.

## Methods

We collected plasma samples from a cohort of healthcare workers who received Pfizer–BioNTech vaccination at our medical center in Southern California^19^. Serological testing for antibodies to the RBD of the S1 subunit of the viral spike protein (IgG (S-RBD)) and antibodies targeting the viral nucleocapsid protein (IgG(N)) was performed at Abbott Labs using the SARS-CoV-2 IgG II assay and SARS-CoV-2 IgG assay, respectively. Antibody levels were measured at three time points: before or up to 3 d after dose 1; within 7–21 d after dose 1; and within 7–21 d after dose 2. We considered an IgG(N) S/C of ≥1.4 as denoting seropositive status due to prior SARS-CoV-2 exposure based on a previously established cutoff^20^.

For assessing potential response of neutralizing antibodies, we considered a conservatively high IgG(S-RBD) threshold of 4,160 AU ml^−1^ as a correlate of neutralization. The 4,160 AU ml^−1^ threshold has been shown to corresponded to a 0.95 probability of obtaining a PRNT ID_50_ at a 1:250 dilution as a representative high titer; thus, we examined the proportion of vaccine recipients who achieved this IgG(S-RBD) threshold^17^ after administration of each dose of vaccine. Furthermore, as a high-throughput method of directly assessing viral inhibition (or neutralization), we also employed a research ACE2 binding inhibition assay to measure the ability of detected IgG(S-RBD) antibodies to inhibit viral spike protein from binding to ACE2 receptors. This ACE2 binding inhibition assay, which we applied to the post-vaccination samples, is known to correlate well with SARS-CoV-2 PRNT methodology and exhibits a high correlation with the IgG(S-RBD) assay threshold employed (*r*^2^ = 0.95).

All participants provided survey data on prior SARS-CoV-2 infection and symptoms experienced after each vaccine dose. We used an online REDCap^21,22^ questionnaire to survey all participants regarding post-vaccination symptoms, including presence or absence of distinct symptom types as well as the severity and duration of any symptom experienced. We administered the questionnaire 8 d after each vaccine dose. We defined a post-vaccine symptom reaction as significant if reported as a non-injection site symptom that was: (1) moderate to severe in degree and lasting <2 d or (2) of any severity and lasting >2 d. We determined prior SARS-CoV-2 infection status and timing in relation to first vaccine date, based on concordance of data documented in health records, presence of IgG(N) antibodies at baseline pre-vaccination testing and the self-reported survey information collected. All cases of data discrepancy regarding prior SARS-CoV-2 infection status underwent manual physician adjudication, including medical chart review for evidence of positive SARS-CoV-2 PCR or antibody testing that was performed by outside institutions.

### Statistical analyses

We compared antibody levels and symptom responses between individuals with and without a prior SARS-CoV-2 infection diagnosis. We analyzed data at both matched and shifted time points, including measures for individuals with a prior infection (at baseline and after dose 1) compared to infection-naive individuals (after dose 1 and dose 2). We log-transformed non-normally distributed values. For comparing between-group continuous values, we used the non-parametric Wilcoxon rank sum test. For comparing between-group proportions (for example, values above a given threshold), we used two-sided chi-square tests with the Yates correction. We performed sensitivity analyses for those individuals with immunoassays at all three time points. We conducted all statistical analyses using R (v3.6.1) and considered statistical significance as a two-tailed *P* value <0.05. All participants provided written informed consent, and all protocols were approved by the Cedars-Sinai institutional review board.

### Reporting Summary

Further information on research design is available in the Nature Research Reporting Summary linked to this article.

## Online content

Any methods, additional references, Nature Research reporting summaries, source data, extended data, supplementary information, acknowledgements, peer review information; details of author contributions and competing interests; and statements of data and code availability are available at 10.1038/s41591-021-01325-6.

## Supplementary information

Supplementary Information Supplementary Tables 1–11.

Reporting Summary

## Data Availability

Requests for de-identified data may be directed to the corresponding authors (J.G.B., S.C. and K.S.) and will be reviewed by the Office of Research Administration at Cedars-Sinai Medical Center before issuance of data sharing agreements. Data limitations are designed to ensure patient and participant confidentiality.
