# Supplementary material for: Antibody responses to the BNT162b2 mRNA vaccine in individuals previously infected with SARS-CoV-2
Source: Nat Med. 2021 Apr 1;27(6):981–4. doi: 10.1038/s41591-021-01325-6 (PMC8205849; doi:10.1038/s41591-021-01325-6)
Supplement: Supplementary file 1 — Supplementary Tables 1–11. [file 41591_2021_1325_MOESM1_ESM.pdf]

nature portfolio

https://doi.org/10.1038/s41591-021-01325-6

Supplementary information

Antibody responses to the BNT162b2 mRNA vaccine in individuals previously infected with SARS-CoV-2

In the format provided by the authors and unedited

SUPPLEMENTARY MATERIAL

Supplementary Table 1. Comparison of antibody levels between participants with and without prior SARS-CoV-2 infection at matched time points. Values are shown as median and interquartile range [IQR], with comparisons performed using two-sided Wilcoxon tests. Sample sizes at baseline, After Dose 1, and After Dose 2 included: N=903, N=490, and N=228 for infection naïve persons; and N=78, N=35, and N=11 for persons with prior infection.

	No Prior Infection	Prior Infection	P value
**Log IgG(S-RBD)**			
Baseline	0.6 [0.2, 1.2]	6.0 [4.6, 6.9]	<0.001
After Dose 1	7.0 [6.3, 7.6]	10.0 [9.2, 10.4]	<0.001
After Dose 2	9.9 [9.4, 10.3]	10.6 [10.3, 10.7]	<0.001
**Log IgG(N)**			
Baseline	-3.9 [-4.6, -3.2]	0.6 [-0.5, 1.2]	<0.001
After Dose 1	-2.8 [-3.9, -1.7]	0.6 [-0.1, 1.2]	<0.001
After Dose 2	-2.7 [-3.7, -1.3]	1.0 [0.2, 1.1]	<0.001
**Log IgM(S)**			
Baseline	-3.2 [-3.5, -2.7]	-0.3 [-1.4, 0.8]	<0.001
After Dose 1	0.1 [-0.8, 0.8]	0.1 [-0.4, 1.0]	0.43
After Dose 2	0.7 [-0.1, 1.3]	-0.1 [-0.6, 1.4]	0.59
**ACE2 binding, %**			
After Dose 1	42.5 [26.1, 58.0]	99.6 [97.4, 100.0]	<0.001
After Dose 2	98.6 [96.9, 99.2]	100.0 [99.0, 100.0]	<0.001

Supplementary Table 2. Comparison of antibody levels between participants with and without prior SARS-CoV-2 infection at shifted time points. Values are shown as median and interquartile range [IQR], with comparisons performed using two-sided Wilcoxon tests. Sample sizes at baseline, After Dose 1, and After Dose 2 included: N=903, N=490, and N=228 for infection naïve persons; and N=78, N=35, and N=11 for persons with prior infection.

	**No Prior Infection**	**Prior Infection**	**P value**
	After Dose 1	***At Baseline***	
Log IgG(S-RBD)	7.0 [6.3, 7.6]	6.0 [4.6, 6.9]	<0.001
Log IgG(N)	-2.8 [-3.9, -1.7]	0.6 [-0.5, 1.2]	<0.001
Log IgM(S)	0.1 [-0.8, 0.8]	-0.3 [-1.4, 0.8]	0.09
	After Dose 2	***After Dose 1***	
Log IgG(S-RBD)	9.9 [9.4, 10.3]	10.0 [9.2, 10.4]	0.92
Log IgG(N)	-2.7 [-3.6, -1.3]	0.6 [-0.1, 1.2]	<0.001
Log IgM(S)	0.7 [-0.1, 1.3]	0.1 [-0.4, 1.0]	0.050
ACE2 binding, %	98.6 [96.9, 99.2]	99.6 [97.4, 100.0]	<0.001

Supplementary Table 3. Sensitivity analysis comparing antibody levels between participants with and without prior SARS-CoV-2 infection at matched time points. Values are shown as median and interquartile range [IQR], with comparisons performed using two-sided Wilcoxon tests. The sample with data available at all time points included N=207 infection naïve persons and N=10 with prior infection.

	No Prior Infection	Prior Infection	P value
**Log IgG(S-RBD)**			
Baseline	0.7 [0.2, 1.2]	5.9 [2.7, 7.2]	<0.001
After Dose 1	7.0 [6.2, 7.6]	10.2 [8.4, 10.5]	<0.001
After Dose 2	9.9 [9.4, 10.3]	10.6 [10.3, 10.8]	0.001
**Log IgG(N)**			
Baseline	-3.9 [-4.6, -3.0]	0.7 [0.1, 1.0]	<0.001
After Dose 1	-2.7 [-3.9, -1.6]	0.8 [-0.1, 1.2]	<0.001
After Dose 2	-2.7 [-3.9, -1.3]	0.9 [0.0, 1.1]	<0.001
**Log IgM(S)**			
Baseline	-3.2 [-3.5, -2.7]	-1.2 [-1.9, 0.5]	<0.001
After Dose 1	0.0 [-0.7, 0.7]	0.1 [-0.2, 1.0]	0.49
After Dose 2	0.7 [-0.1, 1.3]	-0.2 [-0.7, 1.1]	0.24
**ACE2 binding, %**			
After Dose 1	42.1 [26.3, 58.8]	99.9 [77.3, 100.0]	<0.001
After Dose 2	98.6 [96.9, 99.2]	100.0 [99.0, 100.0]	0.001

Supplementary Table 4. Sensitivity analysis comparing antibody levels between participants with and without prior SARS-CoV-2 infection at shifted time points. Values are shown as median and interquartile range [IQR], with comparisons performed using two-sided Wilcoxon tests. The sample with data available at all time points included N=207 infection naïve persons and N=10 with prior infection.

	**No Prior Infection**	**Prior Infection**	**P value**
	After Dose 1	***At Baseline***	
Log IgG(S-RBD)	7.0 [6.2, 7.6]	5.9 [2.7, 7.2]	0.050
Log IgG(N)	-2.7 [-3.9, -1.6]	0.7 [0.1, 1.0]	0.001
Log IgM(S)	0.0 [-0.7, 0.7]	-1.2 [-1.9, 0.5]	0.08
	After Dose 2	***After Dose 1***	
Log IgG(S-RBD)	9.9 [9.4, 10.3]	10.2 [8.4, 10.5]	0.58
Log IgG(N)	-2.7 [-3.9, -1.3]	0.8 [-0.1, 1.2]	<0.001
Log IgM(S)	0.7 [-0.1, 1.3]	0.1 [-0.2, 1.0]	0.31
ACE2 binding, %	98.6 [96.9, 99.2]	99.9 [77.3, 100.0]	0.033

Supplementary Table 5. Comparison of proportions (%) of Anti-Spike Receptor Binding Domain IgG antibody levels ≥4160 AU/mL between participants with and without prior SARS-CoV-2 infection. We use two-sided Chi-Square tests with the Yate's correction to conduct between-group comparisons, without adjustment for multiple testing. Sample sizes at baseline, after dose 1, and after dose 2 included: N=903, N=490, and N=228 for infection naïve persons; and N=78, N=35, and N=11 for persons with prior infection.

	**No Prior Infection**	**Prior Infection**	**P value**
**Matched time points**			
**IgG(S-RBD) ≥4160 AU/mL**	**n/N (%)**	**n/N (%)**	
Baseline	0/903 (0.0%)	6/78 (7.7%)	<0.001
After Dose 1	37/490 (7.6%)	27/35 (77.1%)	<0.001
After Dose 2	221/227 (97.4%)	11/11 (100.0%)	1.00
**Shifted time points**			
	After Dose 1	***At Baseline***	
IgG(S-RBD) ≥4160	37/490 (7.6%)	6/78 (7.7%)	1.00
	After Dose 2	***After Dose 1***	
IgG(S-RBD) ≥4160	221/227 (97.4%)	27/35 (77.1%)	<0.001

Supplementary Table 6. Comparison of proportions (%) of ACE2 binding ≥50% between participants with and without prior SARS-CoV-2 infection. We use two-sided Chi-Square tests with the Yate's correction to conduct between-group comparisons, without adjustment for multiple testing. Sample sizes after dose 1 and after dose 2 included: N=490, and N=228 for infection naïve persons; and N=35, and N=11 for persons with prior infection.

	**No Prior Infection**	**Prior Infection**	**P value**
**Matched time points**			
**ACE binding ≥50%**	**n/N (%)**	**n/N (%)**	
After Dose 1	183/490 (37.3%)	33/35 (94.3%)	<0.001
After Dose 2	223/228 (97.8%)	11/11 (100.0%)	1.00
**Shifted time points**			
	After Dose 2	***After Dose 1***	
ACE binding ≥50%	223/228 (97.8%)	33/35 (94.3%)	0.52

Supplementary Table 7. Significant post-vaccine symptoms by SARS-CoV-2 infection status following dose 1 and dose 2 of mRNA vaccine. We use two-sided Chi-Square tests with the Yate's correction to conduct between-group comparisons, without adjustment for multiple testing. We defined a post-vaccine symptom reaction as significant if reported as a non-injection site symptom that was: (i) moderate to severe in degree and lasting <2 days, or (ii) of any severity and lasting >2 days.

	**No Prior Infection (n=893)**	**Prior Infection (n=76)**	**P value**
After Dose 1, n (%)	223 (25.0%)	28 (36.8%)	0.033
After Dose 2, n (%)	524 (58.7%)	39 (51.3%)	0.259

Supplementary Table 8. Significant post-vaccine symptoms following first and second mRNA vaccine doses by SARS-CoV-2 infection status. We use two-sided Chi-Square tests with the Yate's correction to conduct between-group comparisons, without adjustment for multiple testing. We defined a post-vaccine symptom reaction as significant if reported as a non-injection site symptom that was: (i) moderate to severe in degree and lasting <2 days, or (ii) of any severity and lasting >2 days.

	No Prior Infection After Dose 2 **(n=893)**	***Prior Infection After Dose 1*** **(n=76)**	**P value**
Reactogenicity, n (%)	524 (58.7%)	28 (36.8%)	<0.001

Supplementary Table 9. Significant post-vaccine symptoms by dose (first and second) of mRNA vaccine stratified by prior SARS-CoV-2 infection status. We use two-sided Chi-Square tests with the Yate's correction to conduct between-group comparisons, without adjustment for multiple testing. We defined a post-vaccine symptom reaction as significant if reported as a non-injection site symptom that was: (i) moderate to severe in degree and lasting <2 days, or (ii) of any severity and lasting >2 days.

	**After Dose 1 (n=839)**	**After Dose 2 (n=76)**	**P value**
No Prior Infection, n (%)	223 (25.0%)	524 (58.7%)	<0.001
Prior Infection, n (%)	28 (36.8%)	39 (51.3%)	0.082

Supplementary Table 10. Types of symptoms reported following vaccine dose 1, by prior SARS-CoV-2 infection status. We use two-sided Chi-Square tests with the Yate's correction to conduct between-group comparisons, without adjustment for multiple testing.

	No Prior Infection (n=893)	Prior Infection (n=76)	P value
**Symptom, n (%)**			
Fatigue or Malaise	138 (15.5%)	15 (19.7%)	0.41
Headache, Dizziness or Lightheadedness	105 (11.8%)	9 (11.8%)	1.00
Fever or Chills	35 (3.9%)	10 (13.2%)	0.001
Muscle, Bone, Joint or Nerve Symptoms	69 (7.7%)	10 (13.2%)	0.15
Nausea, Vomiting, Diarrhea or Other Digestive Symptoms	13 (1.5%)	2 (2.6%)	0.75
Sleep Changes	17 (1.9%)	3 (3.9%)	0.43
Swollen Lymph Nodes, Skin, Nail or Facial Changes	6 (0.7%)	1 (1.3%)	1.00
Eye, Ear, Mouth or Throat Changes	13 (1.5%)	1 (1.3%)	1.00
Cough, Chest or Breathing Symptoms	10 (1.1%)	1 (1.3%)	1.00
Memory of Mood Changes	7 (0.8%)	1 (1.3%)	1.00

Supplementary Table 11. Types of symptoms reported following dose 2, by prior SARS-CoV-2 infection status. We use two-sided Chi-Square tests with the Yate's correction to conduct between-group comparisons, without adjustment for multiple testing.

	No Prior Infection (n=893)	Prior Infection (n=76)	P value
**Symptom, n (%)**			
Fatigue or Malaise	412 (46.1%)	31 (40.8%)	0.44
Headache, Dizziness or Lightheadedness	274 (30.7%)	13 (17.1%)	0.018
Fever or Chills	254 (28.4%)	22 (28.9%)	1.00
Muscle, Bone, Joint or Nerve Symptoms	215 (24.1%)	18 (23.7%)	1.00
Nausea, Vomiting, Diarrhea or Other Digestive Symptoms	67 (7.5%)	5 (6.6%)	0.95
Sleep Changes	77 (8.6%)	5 (6.6%)	0.69
Swollen Lymph Nodes, Skin, Nail or Facial Changes	41 (4.6%)	1 (1.3%)	0.29
Eye, Ear, Mouth or Throat Changes	16 (1.8%)	2 (2.6%)	0.94
Cough, Chest or Breathing Symptoms	16 (1.8%)	0 (0.0%)	0.48
Memory of Mood Changes	21 (2.4%)	3 (3.9%)	0.64
