# Supplementary material for: Antibody responses to the BNT162b2 mRNA vaccine in individuals previously infected with SARS-CoV-2
Source: Nat Med. 2021 Apr 1;27(6):981–4. doi: 10.1038/s41591-021-01325-6 (PMC8205849; doi:10.1038/s41591-021-01325-6)
Supplement: Supplementary file 2 — Reporting Summary [file 41591_2021_1325_MOESM2_ESM.pdf]

nature research

Corresponding author(s): Kimia Sobhani, PhD; Susan Cheng, MD, MPH; Jonathan G. Braun, MD, PhD

Last updated by author(s): Mar 15, 2021

Reporting Summary

Nature Research wishes to improve the reproducibility of the work that we publish. This form provides structure for consistency and transparency in reporting. For further information on Nature Research policies, see our Editorial Policies and the Editorial Policy Checklist.

Statistics

For all statistical analyses, confirm that the following items are present in the figure legend, table legend, main text, or Methods section.

n/a	Confirmed	
☐	☒	The exact sample size (*n*) for each experimental group/condition, given as a discrete number and unit of measurement
☐	☒	A statement on whether measurements were taken from distinct samples or whether the same sample was measured repeatedly
☐	☒	The statistical test(s) used AND whether they are one- or two-sided<br>*Only common tests should be described solely by name; describe more complex techniques in the Methods section.*
☐	☒	A description of all covariates tested
☐	☒	A description of any assumptions or corrections, such as tests of normality and adjustment for multiple comparisons
☒	☐	A full description of the statistical parameters including central tendency (e.g. means) or other basic estimates (e.g. regression coefficient) AND variation (e.g. standard deviation) or associated estimates of uncertainty (e.g. confidence intervals)
☐	☒	For null hypothesis testing, the test statistic (e.g. *F*, *t*, *r*) with confidence intervals, effect sizes, degrees of freedom and *P* value noted<br>*Give P values as exact values whenever suitable.*
☒	☐	For Bayesian analysis, information on the choice of priors and Markov chain Monte Carlo settings
☒	☐	For hierarchical and complex designs, identification of the appropriate level for tests and full reporting of outcomes
☒	☐	Estimates of effect sizes (e.g. Cohen's *d*, Pearson's *r*), indicating how they were calculated

Our web collection on statistics for biologists contains articles on many of the points above.

Software and code

Policy information about availability of computer code

Data collection	No custom algorithms or software used.
Data analysis	All statistical analyses were conducted using R v3.6.1 and no custom code packages were used. All code used for analyses is available upon request.

For manuscripts utilizing custom algorithms or software that are central to the research but not yet described in published literature, software must be made available to editors and reviewers. We strongly encourage code deposition in a community repository (e.g. GitHub). See the Nature Research guidelines for submitting code & software for further information.

Data

Policy information about availability of data

All manuscripts must include a data availability statement. This statement should provide the following information, where applicable:

- Accession codes, unique identifiers, or web links for publicly available datasets
- A list of figures that have associated raw data
- A description of any restrictions on data availability

Requests for deidentified data may be sent to the corresponding author and are reviewed by Cedars-Sinai Medical Center prior to issuance of data sharing agreements. The data are not publicly available due to the contents containing information that could compromise participant privacy.

Field-specific reporting

Please select the one below that is the best fit for your research. If you are not sure, read the appropriate sections before making your selection.

☒ Life sciences ☐ Behavioural & social sciences ☐ Ecological, evolutionary & environmental sciences

For a reference copy of the document with all sections, see nature.com/documents/nr-reporting-summary-flat.pdf

Life sciences study design

All studies must disclose on these points even when the disclosure is negative.

Sample size	We studied data collected from a total of N=1090 vaccine recipients who provided at least one blood sample for antibody testing and represented the entirety of a cohort recruited via open enrollment. Formal statistical power estimates were not performed, as the sampling strategy was based on maximal open enrollment capacity available at vaccination sites.
Data exclusions	No data were excluded from analyses.
Replication	No separate cohorts of comparable size are currently available for replication, to our knowledge.
Randomization	Randomization was not relevant to this experiment, as this was an observational study.
Blinding	Blinding was not relevant because of the observational study design (i.e. examining the differences in antibody levels between individuals previously identified as with versus without prior COVID-19).

Reporting for specific materials, systems and methods

We require information from authors about some types of materials, experimental systems and methods used in many studies. Here, indicate whether each material, system or method listed is relevant to your study. If you are not sure if a list item applies to your research, read the appropriate section before selecting a response.

Materials & experimental systems

n/a	Involved in the study	
☐	☒	Antibodies
☒	☐	Eukaryotic cell lines
☒	☐	Palaeontology and archaeology
☒	☐	Animals and other organisms
☐	☒	Human research participants
☒	☐	Clinical data
☒	☐	Dual use research of concern

Methods

n/a	Involved in the study	
☒	☐	ChIP-seq
☒	☐	Flow cytometry
☒	☐	MRI-based neuroimaging

Antibodies

Antibodies used

Abbott Architect immunoassays (SARS-CoV-2 IgG and SARS-CoV-2 IgG II) from Abbott Labs were used to quantify circulating levels of SARS-CoV-2 anti-nucleocapsid (N) and anti-spike (S) antibodies.

Abbott SARS-CoV-2 IgG II assay uses the following for IgG(S-RBD):
Paramagnetic Microparticles: Purified SARS-CoV-2 recombinant S-RBD antigen coated microparticles in TRIS buffer with surfactant.
Conjugate Antibody: Anti-human IgG (mouse, monoclonal) acridinium-labeled conjugate in MES buffer with surfactants and protein (bovine) stabilizer.

The Abbott SARS-CoV-2 IgG assay uses the following for IgG(N):
Paramagnetic Microparticles: Purified SARS-CoV-2 recombinant N antigen coated microparticles in TRIS buffer with surfactant.
Conjugate Antibody: Anti-human IgG (mouse, monoclonal) acridinium-labeled conjugate in MES buffer with surfactant and protein (bovine) stabilizer.

The ACE2 binding inhibition assay involves S-RBD coated magnetic microparticles (as for the IgG II quant assay) and a recombinant acridinium-labeled ACE2, and the signal decreases as ACE2 binding to S-RBD coated microparticles is reduced due to the presence of sample-specific neutralizing antibodies.

Validation

Abbott SARS-CoV-2 immunoassays with validation reported in J Clin Microbiol. 2020;58(8):e00941-20.

Human research participants

Policy information about studies involving human research participants

Population characteristics	A diverse and unselected population of adults employed in a multi-site healthcare delivery system located in Los Angeles County, including individuals with direct patient contact and others with non-patient-oriented work functions. Our study sample is representative of the institution's diverse catchment area of 1.8 million, approximately half of whom identify as a racial or ethnic minority. Full cohort characteristics are outlined in Table 1 of the manuscript.
Recruitment	Recruitment via newsletters and fliers, open to any Cedars-Sinai employees approved and scheduled for vaccine. Enrollment was not otherwise restricted based on any pre-specified criteria. We anticipate that our study results regarding the antibody response to vaccination is not unique to the characteristics of the healthcare workers who enrolled in this study and that, in turn, any unintended sampling biases would not materially impact the generalizability of results.
Ethics oversight	Cedars-Sinai Institutional Review Board

Note that full information on the approval of the study protocol must also be provided in the manuscript.
